# Anthracycline and concurrent radiotherapy as adjuvant treatment of operable breast cancer: a retrospective cohort study in a single institution

**DOI:** 10.1186/1756-0500-3-247

**Published:** 2010-10-04

**Authors:** Nabil Ismaili, Sanaa Elmajjaoui, Issam Lalya, Lamia Boulaamane, Rhizlane Belbaraka, Halima Abahssain, Rachi Aassab, Noureddine Benjaafar, Brahim El Khalil El Guddari, Omar El Mesbahi, Yassir Sbitti, Mohammed Ismaili, Hassan Errihani

**Affiliations:** 1 Department of Medical Oncology, National Institute of Oncology, Rabat, Morocco; 2 Department of Medical Oncology, Regional Cancer Center, Agadir, Morocoo; 3 Department of Radiation Oncology, National Institute of Oncology, Rabat, Morocco; 4 Department of Medical Oncology, Hassan II Hospital, Fes, Morocco; 5 Department of Medical Oncology, Mohammed V Military Hospital, Rabat, Morocco; 6 Department of Microbiology, Moulay Ismail University, Meknes, Morocco

## Abstract

**Background:**

Concurrent chemoradiotherapy (CCRT) after breast surgery was investigated by few authors and remains controversial, because of concerns of toxicity with taxanes/anthracyclines and radiation. This treatment is not standard and is more commonly used for locally advanced breast cancer. The aim of our study was to evaluate the efficacy and safety of the concomitant use of anthracycline with radiotherapy (RT).

**Findings:**

Four hundred women having operable breast cancer, treated by adjuvant chemotherapy (CT) and RT in concomitant way between January 2001 and December 2003, were included in this retrospective cohort study. The study compares 2 adjuvant treatments using CCRT, the first with anthracycline (group A) and the second with CMF (group B). The CT treatment was repeated every 21 days for 6 courses and the total delivered dose of RT was 50 Gy, divided as 2 Gy daily fractions. Locoregional recurrence free (LRFS), event free (EFS), and overall survivals (OS) were estimated by the Kaplan-Meier method. The log-rank test was used to compare survival events. Multivariate Cox-regression was used to evaluate the relationship between patient characteristics, treatment and survival.

In the 2 groups (A+B) (n = 400; 249 in group A and 151 in group B), the median follow-up period was 74.5 months. At 5 years, the isolated LRFS was significantly higher in group A compared to group B (98.7% vs 95.3%; hazard ratio [HR] = 0.258; 95% CI, 0.067 to 0.997; log-rank *P *= .034). In addition, the use of anthracycline regimens was associated with a higher rate of 5 years EFS (80.4% vs 75.1%; HR = 0.665; 95% CI, 0.455 to 1.016; log-rank *P *= .057). The 5 years OS was 83.2% and 79.2% in the anthracycline and CMF groups, respectively (HR = 0.708; 95% CI, 0.455 to 1.128; log-rank *P *= .143). Multivariate analysis confirmed the positive effect of anthracycline regimens on LRFS (HR = 0.347; 95% CI, 0.114 to 1.053; log-rank *P *= .062), EFS (HR = 0.539; 95% CI, 0.344 to 0.846; *P *= 0.012), and OS (HR = 0.63; 95% CI, 0.401 to 0.991; *P *= .046). LRFS, EFS and OS were significantly higher in the anthracycline group where the patients (n = 288) received more than 1 cycle of concurrent CT (*P *= .038, *P *= .026 and *P *= .038, respectively). LRFS and EFS were significantly higher in the anthracycline group within the BCT subgroup (*P *= .049 and *P *= .04, respectively). There were more hematologic, and more grade 2/3/4 skin toxicity in the anthracycline group.

**Conclusions:**

After mastectomy or BCT, the adjuvant treatment based on anthracycline and concurrent RT reduced breast cancer relapse rate, and significantly improved LRFS, EFS and OS in the patients receiving more than 1 cycle of concurrent CT. There were more hematologic and non hematologic toxicities in the anthracycline group.

## Background

In the case of radical mastectomy or breast conservative therapy (BCT), adjuvant radiotherapy (RT), improves local control [1-9]. Adjuvant chemotherapy (CT) is equally mandatory for diminishing metastatic recurrences [10-12].

The optimal sequence of CT and RT remains controversial. The delivery of RT can be planned before or after CT, concurrently, or within 2 cycles of CT (''sandwich'' therapy). However, the current standard is CT followed by whole-breast-irradiation (WBI). The delay of CT increased the incidence of spared metastasis [13-16], and the delay of RT induced more frequent local recurrences [17-19].

Concurrent treatment shortens the duration of therapy, allows RT and CT to start temporally, and potentially improves local control via the radiation sensitizing effects of CT. Concurrent chemoradiotherapy (CCRT) has successfully been achieved with cyclophosphamide, methotrexate and fluorouracil (CMF) [20-22]. The concurrent use of taxanes and WBI seems feasible [23].

Anthracycline regimens improve survival in the adjuvant setting [24]. Unfortunately, concomitant use of epirubicin or doxorubicin produced 30% to 44% rate of high grade radiation dermatitis (RD) [25,26], and concurrent mediastinal irradiation with doxorubicin induced intense cardiac dysfunction [27]. This was discouraging the use of anthracyclines and radiation concurrently.

However, in a previous study conducted in our institute we showed that CCRT using anthracycline and WBI produced only 4.5% of ≥ grade 2 RD, and improved the locoregional control over the treatment based on CMF [28]. And recently, in another study, the authors confirmed the feasibility of partial breast irradiation (PBI) with concurrent dose-dense doxorubicin and cyclophosphamide in early breast cancer [29].

The present work, by including more patients, had the objective to confirm our previous results and to support the feasibility and efficiency of concomitant use of anthracycline [28,30]. It was based on the study of a data base of 400 patients treated by radical surgery (mastectomy or breast conservative therapy [BCT]) and adjuvant CCRT. We compared the efficacy, and tolerability of two concomitant protocols, the first with anthracycline based regimens and the second with CMF regimen.

## Methods

### 1. Patient selection

#### Eligibility

From January 2001 to December 2003, 400 women with pathological confirmed breast cancer at stages pT1-T4/pN0-3, without any evidence of metastatic disease, who received complete excision of the primary tumor (mastectomy or BCT) and treated with adjuvant CCRT, were selected at the National Institute of Oncology in Rabat, for investigation.

Two hundred forty four of the cases analyzed in the present study have been the subject of one previous published report [28].

Patient medical records (demographic data, disease stages, histological findings, treatment and outcome) were analyzed retrospectively. Radiological, pathological and surgical reports were reviewed to determine the stage of the disease [31]. The diagnostic instrumental examinations used to stage the patients were chest radiograph, abdominal ultrasound performed in all patients, and bone scan (performed in only 15.2% of the patients [n = 61]).

#### Exclusion criteria

Metastatic breast cancer or incomplete excision of the primary tumour, and patients treated with sequential chemo-radiotherapy.

### 2. Treatment planning (Figure 1)

**Figure 1:**
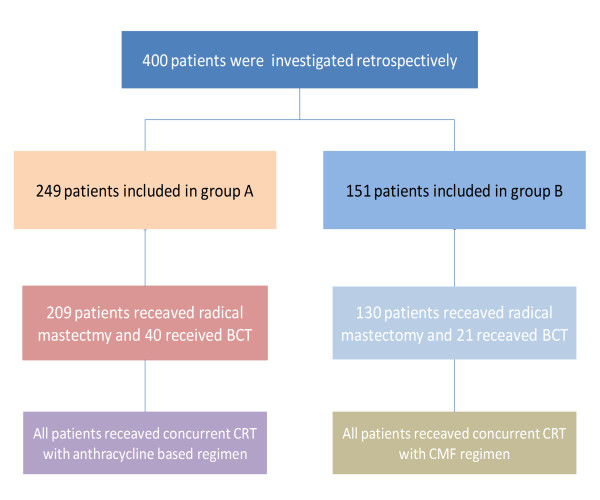
**Study design**. BCT = breast conservative therapy.

The patients from which 84.8% (n = 361) had radical surgery and 15.2% (n = 61) had BCT, were divided into two groups on the basis of CT treatment. In group A the treatment was based on anthracycline and in group B the treatment was based on CMF. The median number of CT cycles delivered concurrently with RT was 2 (ranging from 1 to 5).

Group A (n = 249) received CCRT with anthracycline based regimen and group B (n = 151) received CCRT with CMF regimen.

#### Radiotherapy

For 96.5% of patients, RT was delivered to the whole breast or to thoracic wall (99.2% in group A and 92.1% in group B). The other 3.5% received only RT in the lymph node region, this can be explained by the fact that these patients had small tumors (pT1-pT2), and received mastectomy with inadequate axillary dissection. 93.3% of the patients received an additional treatment RT delivered to the regional lymph nodes.

All patients were treated with external beam RT using tangential fields of Co- 60-gamma-Ray. The total delivered dose was 50 Gy, divided as 2 Gy daily fractions. A complementary treatment was given by electrons or by breast brachytherapy (10 to 20 Gy) for 28 patients.

#### Chemotherapy

The rational of choice of CT was not precised in the patient files. The use of anthracycline regimen was justified by more aggressive tumors and by the lack of availability of other CT drugs.

Chemotherapy consisted of:

- In group A: a- intravenous AC60 (doxorubicin 60 mg/m^2 ^and cyclophosphamide 600 mg/m^2^) on day 1, repeated every 21 days for six courses for 173 patients, b- intravenous FEC75 (5-fluorouracile 500 mg/m^2^, epirubicin 75 mg/m^2^, and cyclophosphamide 500 mg/m^2^) on day 1, repeated every 21 days for six courses for 37 patients and c- intravenous FAC50 (5-fluorouracile 500 mg/m^2^, doxorubicin 50 mg/m^2^, and cyclophosphamide 500 mg/m^2^) on day 1, repeated every 21 days for six courses for 26 patients, and d- Thirteen patient received sequential treatment based on 2 different regimens, the first based on anthracycline and the second based on CMF, repeated every 21 days for six courses (table S1 [additional file 1]). The timing of CCRT was summarized in table S1 (additional file 1).

- In group B: intravenous CMF (cyclophosphamide 500 mg/m^2^, methotrexate 60 mg/m^2^, and 5-fluorouracil 500 mg/m^2^) on day 1, repeated every 21 days for six courses for 151 patients.

#### Hormone therapy

Adjuvant 5-year tamoxifen was initiated at the end of the CCRT treatment in patients having positive hormone receptor status.

### 3. Study endpoints

We retrospectively compared locoregional recurrence free survival (LRFS), event free survival (EFS), overall survival (OS), and toxicity between 2 therapeutic groups A and B and between the subgroups within A and B. The primary outcomes were LRFS and EFS, and the secondary outcomes were OS and safety.

### 4. Toxicity evaluation

The hematologic toxicity was measured through laboratory tests. Only the highest grades of toxicity were considered in the analysis.

Information about non-hematologic toxicities (dermatitis toxicity, cardiac toxicity, and pulmonary toxicity) was not routinely collected. Only few high grades toxicities (≥grade 2) were noted in our data base. This constitutes a major limitation of our retrospective study.

### 5. Statistical analysis

LRFS, EFS and OS were calculated from the date of diagnosis (fine needle aspiration, biopsy) or surgery to the date of first documented locoregional relapse, and/or to the date of death and last follow-up.

LRFS was of 2 types: in type 1, the events considered were locoregional and occurred in the breast and/or chest wall and/or regional lymph nodes, with or without concurrent metastatic recurrence; in type 2 (isolated LRFS), the events occurred concurrently with metastatic recurrence were not considered.

The Kaplan-Meier method was used to estimate the rates of LRFS, EFS, and OS [32]. The log-rank-test was used to evaluate the differences between the groups. The distribution homogeneity was analyzed with the Pearson-chi^2^-test for both groups. The p values were based on two sided tests and conducted at a 5% significance level

Univariate and multivariate univariate Cox proportional hazard regression models analysis was used to evaluate the relationship between survival (LRFS, EFS, and OS), treatment regimen (anthracycline vs CMF), and patient characteristics (age, lymph node involvement, tumour volume, tumour grade, hormonal receptor status, number of cycle of concurrent CT) [33]. Candidate prognostic factors for LRFS, EFS, and OS, with a 0.5 level of significance in univariate analysis were entered in a multivariate Cox model.

Statistical evaluation was carried out using SPSS 17.0 statistical software.

### 6. Subgroup Analyses

The first subgroup concerned the patients who received ≥ 2 cycles of concurrent CT (n = 288), and the second subgroup concerned the patients treated with BCT (n = 61).

### 7. Consent and statement of ethical approval

As the treatment was decided by the medical staff of the centre depending on the availability of drugs in Morocco, oral consent was obtained from the subjects and was approved by the institutional review boards of the National Institute of Oncology Cancer Centre in Rabat

This study was approved by the institutional review boards of the National Institute of Oncology Cancer Centre in Rabat.

This research is in compliance with the Helsinki Declaration

## Results

### 1. Patient characteristics

Median age was 44 years (range: 22 - 69 years) in group A and 46 years (26 - 95 years) in group B.

The distribution of patient characteristics was partly imbalanced. After the analysis of characteristics homogeneity within the two groups, we found more women aged less than 40 years (*P *= .001), more lymph node involvement (*P *= .019) in group A than in group B, and less patients were menopaused in group A. In addition, we found in group B that more patients received more than 1 cycle of CT concurrently with RT (*P *< .001) (table 1).

**Table 1:** Demographic, clinical, histological, molecular and treatment characteristics of all patients (n = 400) and analysis of groups homogeneity (Pearson Chi^2^-test)

Characteristics	Group A [n = 249] No (%)	Group B [n = 151] No (%)	*P *value
**Age**			
**< 40**	76 (30.5%)	25 (16.6%)	0.001
**≥ 40**	173 (69.5%)	122 (83.4%)	
**Menopausal status**			
**No**	178 (71.5%)	85 (56.3%)	0.008
**Yes**	60 (24.1%)	56 (37.1%)	
**Unknown**	11 (4.4%)	10 (6.6%)	
**Side**			
**Right**	128 (51.4%)	72 (47.7%)	0.552
**Left**	120 (48.2%)	79 (52.2%)	
**Bilateral**	1 (0.4%)	0	
**Surgery**			
**Mastectomy**	209 (83.9%)	130 (86.1%)	0.561
**BCT**	40 (16.1%)	21 (13.9%)	
**Histology**			
**DIC**	231 (92.8%)	136 (90.1%)	0.248
**LIC**	12 (4.8%)	13 (8.6%)	
**Other**	1	0	
**Unknown**	5 (2%)	2 (1.3%)	
**SBR**			
**I**	15 (6%)	12 (7.9%)	0.72
**II**	153 (61.4%)	97 (64.2%)	
**III**	74 (29.7%)	38 (25.2%)	
**Unknown**	7 (2.8%)	4 (2.6%)	
**Hormonal Receptor**			
**ER+/PR+**	123 (49.4%)	84 (55.6%)	0.205
**ER+/PR-**	31 (12.4%)	25 (16.6%)	
**ER-/PR+**	25 (10%)	12 (7.9%)	
**ER-/PR-**	65 (26.1%)	28 (18.5%)	
**Unknown**	5 (2%)	2 (1.3%)	
**Tumour**			
**pT1**	28 (11.2%)	21 (13.9%)	0.867
**pT2**	142 (57%)	84 (55.6%)	
**pT3**	63 (25.3%)	35 (23.2%)	
**pT4**	12 (4.8%)	7 (4.6%)	
**Unknown**	4 (1.6%)	4 (2.6%)	
**pN, axillary**			
**pN0**	44 (17.7%)	42 (27.8%)	0.009
**pN1**	69 (27.7%)	46 (30.5%)	
**pN2**	77 (30.9%)	47 (31.1%)	
**pN3**	50 (20.1%)	13 (8.6%)	
**Unknown**	9 (3.6%)	3 (2%)	
**Number of cycle of CT delivered with RT**			
**1**	87 (34.9%)	25 (16.6%)	< 0.001
**≥ 2**	162 (65.1%)	126 (83.4%)	
**Breast/thoracic wall irradiation**			
**No**	2 (0.8%)	12 (7.9%)	< 0.001
**Yes**	247 (99.2%)	139 (92.1%)	
**Prophylactic supraclavicular fossa radiotherapy**			
**No**	17 (6.8%)	10 (6.6%)	0.556
**Yes**	232 (93.2%)	141 (93.4%)	
**Internal mammary radiotherapy**			
**No**	16 (6.4%)	9 (6%)	0.517
**Yes**	233 (93.6%)	142 (94%)	
**Axillary radiotherapy**			
**No**	220 (88.4%)	120 (79.5%)	0.012
**Yes**	29 (11.6%)	31 (20.5%)	

Abbreviations: BCT = breast conservative therapy; SBR = Scarf-Bloom-Richardson; DIC = ductal invasive carcinoma; LIC = lobular invasive carcinoma; ER = estrogen receptor; PR = progesterone receptor

The analysis of characteristics homogeneity within the subgroup of patients (n = 288) treated with ≥ 2 cycles of concurrent CT showed that more patients in the anthracycline subgroup were younger than 40 (*P *= .044) and there was more lymph nodes involvement (*P *= .009) within anthracycline subgroup (table S2 [additional file 2]).

Sixty one patients were treated with BCT, 40 of them received anthracycline based CT and the remaining 21 received CMF. The characteristics homogeneity was also analyzed between theses small subgroups (table S3 [additional file 3]).

The median delay of CT after surgery was 6.8 weeks (range: 1 to 60 weeks), and the median delay of RT after surgery was 14.8 weeks (range: 2 to 60 weeks). In the 2 groups A and B respectively, 93.6% and 98% of the patients received the 6 courses of CT. All patients in the two groups received 100% of the planned RT dose.

### 2. Treatment compliance

The hematologic toxicity was determined in 394 cases. The percentage of patients which developed grade 2-3 anemia was 10.1% vs 6.1% in group A and B respectively (chi^2^-test, *P *= .172). Grade 3-4 neutropenia was present in 14.6% of the cases in group A vs 7.6% of cases in group B (chi^2^-test, *P *= .036). Grade ≥ 2 thrombopenia was found in 1.2% of the cases in group A vs 0.7% of the cases in group B (chi^2^-test, *P *= .6).

There was no cardiac toxicity that was clinically detectable in the two arms. The left ventricular fraction ejection (LVFE) was evaluated in only 9 patients (2 patients in the anthracycline group and 7 in the CMF group), and was normal (LVFE ranged between 63% and 87%).

In group A (n = 249), 12 patients (4.8%) had ≥ grade 2 RD toxicity, vs 2 patients (1.3%) in group B (n = 151) (chi^2^-test, *P *= .065).

Six patients in the anthracycline group showed respiratory symptoms (dry cough in 5 patients and chest pain in 1 patient) vs 0 patients in the CMF group (chi^2^-test, *P *= .055).

### 3. Outcomes

#### 3.1 Outcomes in group A plus group B (n = 400)

##### LRFS (type 1)

**Figure 2:**
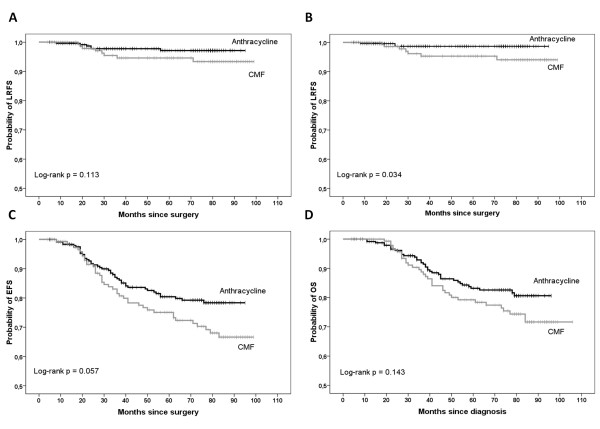
**Comparison of the anthracycline group (group A, n = 249) with the CMF group (group B, n = 151) performed in the whole group of patients (n = 400)**: Kaplan-Meier estimates of LRFS when all locoregional relapses were considered (Panel A), LRFS when only the isolated locoregional relapses were considered (Panel B), EFS (Panel C), and OS (Panel D). LRFS = locoregional recurrence free survival; EFS = event free survival; OS = overall survival.

After 72.9 months (72.8 for group A, and 73.3 for group B) median follow-up (range: 4.5 - 99 months), loco-regional relapse was developed by 6 patients in the anthracycline group and by 8 patients in the CMF group. The 5 years LRFS rate was equal to 97.1% in group A *vs *94.6% in group B (hazard ratio [HR] = 0.454; 95% CI, 0.157 to 1.308; log-rank *P *= .133) (Figure 2A).

##### Isolated LRFS (type 2)

When only isolated locoregional events were considered for the evaluation of LRFS, loco-regional relapse was developed by 3 patients in anthracycline group and by 7 patients in the CMF group. The 5 years LRFS rate was significantly higher in group A (98.7%) than group B *(*95.1%) (HR = 0.258; 95% CI, 0.067 to 0.997; log-rank *P *= .034) (Figure 2B).

##### Event free survival

For EFS, the median follow-up period was 71.2 (72.4 in group A and 64.5 in group B) months (range: 4.5 - 99 months). EFS was evaluated in group A + group B, 86 of whom had experienced an event (relapse or death) at the last follow-up (in anthracycline group: 45 of 249; in CMF group: 41 of 151). The 5 years EFS rate was 80.4% in group A *vs *75.1% in group B, a difference which tended to significance (HR = 0.665; 95% CI, 0.455 to 1.016; log-rank *P *= .057) (Figure 2C).

##### Overall survival

For OS, the median follow-up period was 74.5 (74.5 for group A and 74.6 for group B) months (range, 4.5 - 106.6 months). Seventy-two patients died, 38 in group A and 34 in group B. The 5 years OS rate was 83.2% in group A vs 79.2% in group B, a difference that was not statistically significant (HR = 0.708; 95% CI, 0.455 to 1.128; log-rank *P *= .143) (Figure 2D).

##### Prognostic factors (table 2)

**Table 2:** Factors influencing LRFS, EFS and OS (Cox proportional Hazard Model)

Analysis using locoregional recurrence free survival (type 1)						
**Factors**	**Univariate analysis**			**Multivariate analysis**		
	**HR**	**95% CI**	***P *value**	**HR**	**95% CI**	***P *value**

**Lymph node involvement: no vs yes**	0.794	0.221 to 2.846	0.717	-	-	-
**Tumour: pT1-2 vs pT3-4**	0.273	0.095 to 0.787	0.016	0.326	0.11 to 0.963	0.043
**SBR grade: 1-2 vs 3**	0.692	0.232 to 2.066	0.509	-	-	-
**ER status: positive vs negative**	0.359	0.125 to 1.036	0.058	0.387	0.13 to 1.157	0.079
**Age: ≥ 40 vs < 40**	1.288	0.359 to 4.618	0.697	-	-	-
**Regimen: anthracycline vs CMF**	0.454	0.157 to 1.308	0.143	0.347	0.114 to 1.053	0.062
**Number of cycles of CT delivered concurrently with RT: ≥ 2 vs 1**	0.466	0.223 to 1.987	0.439	0.587	0.244 to 1.414	0.235

**Analysis using event free survival**						

	**Univariate analysis**			**Multivariate analysis**		
	**HR**	**95% CI**	***P *value**	**HR**	**95% CI**	***P *value**

**Lymph node involvement: no vs yes**	0.406	0.216 to 0.766	0.004	0.397	0.216 to 0.779	0.006
**Tumour: pT1-2 vs pT3-4**	0.552	0.356 to 0.855	0.007	0.624	0.392 to 0.993	0.047
**SBR grade: 1-2 vs 3**	0.801	0.508 to 1.265	0.341	0.971	0.598 to 1.576	0.906
**ER status: positive vs negative**	0.674	0.438 to 1.038	0.058	0.64	0.404 to 1.015	0.058
**Age: ≥ 40 vs < 40**	1.183	0.717 to 1.951	0.511	-	-	-
**Regimen: anthracycline vs CMF**	0.665	0.435 to 1.016	0.057	0.558	0.354 to 0.879	0.012
**Number of cycles of CT delivered concurrently with RT: ≥ 2 vs 1**	0.696	0.445 to 1.089	0.11	0.625	0.39 to 1.002	0.051

**Analysis using overall survival**						

	**Univariate analysis**			**Multivariate analysis**		
	**HR**	**95% CI**	***P *value**	**HR**	**95% CI**	***P *value**

**Lymph node involvement: no vs yes**	0.298	0.137 to 0.651	0.002	0.317	0.144 to 0.697	0.004
**Tumour: pT1-2 vs pT3-4**	0.619	0.381 to 1.005	0.052	0.719	0.429 to 1.208	0.213
**SBR grade: 1-2 vs 3**	0.694	0.427 to 1.130	0.142	0.781	0.466 to 1.309	0.347
**ER status: positive vs negative**	0.737	0.458 to 1.186	0.208	0.714	0.429 to 1.189	0.196
**Age: ≥ 40 vs < 40**	1.260	0.723 to 2.197	0.414	-	-	-
**Regimen: anthracycline vs CMF**	0.708	0.445 to 1.128	0.143	0.603	0.367 to 0.99	0.046
**Number of cycles of CT delivered concurrently with RT: ≥ 2 vs 1**	0.768	0.468 to 1.261	0.297	0.713	0.423 to 1.201	0.203

Abbreviations: HR = hazard ratio; CI = confidence interval; SBR = Scarf-Bloom-Richardson; ER = estrogen receptor; CT = chemotherapy; RT = radiotherapy

The proportional hazard model was used because of the non homogeneity of patient characteristics between the two groups A and B, to identify the positive treatment effect of one group over the other, on LRFS (type 1), EFS and OS.

##### -Analysis of prognostic factors using LRFS (type 1)

Univariate analysis showed that the factors influencing LRFS were the tumor size (*P *= .016), with trend toward significance for the ER status (*P *= 0.058) and CT protocol (anthracycline vs CMF) (*P *= .143). Multivariate analysis showed that the factors influencing LRFS were the tumor size (*P *= .043), and trend toward significance for CT protocol (HR = 0.347; 95% CI, 0.114 to 0.1.053; *P *= .062), and ER status (*P *= .079).

##### -Analysis of prognostic factors using EFS

Univariate analysis showed that the factors influencing EFS were the lymph node involvement status (*P *= .004), tumor size (*P *= .007), and trend toward significance for CT protocol (anthracycline vs CMF) (*P *= .057). Multivariate analysis showed that the factors influencing EFS were the lymph node involvement status (*P *= .005), CT protocol (HR = 0.539; 95% CI, 0.344 to 0.846; *P *= 0.012), and tumor size (*P *= .047), trend toward significance for ER status (*P *= .058) and number of cycles delivered concurrently with RT (*P *= .051).

##### -Analysis of prognostic factors using OS

Univariate analysis showed that the factors influencing OS were lymph node status (*P *= .002) and trend toward significance for tumor size (*P *= .052). Multivariate analysis showed that the factors influencing OS was the lymph node status (*P *= .004) and CT protocol (HR = 0.63; 95% CI, 0.401 to 0.991; *P *= .046).

#### 3.2 Outcomes in patients who received more than 1 cycle of CT concurrently with RT (n = 288)

**Figure 3:**
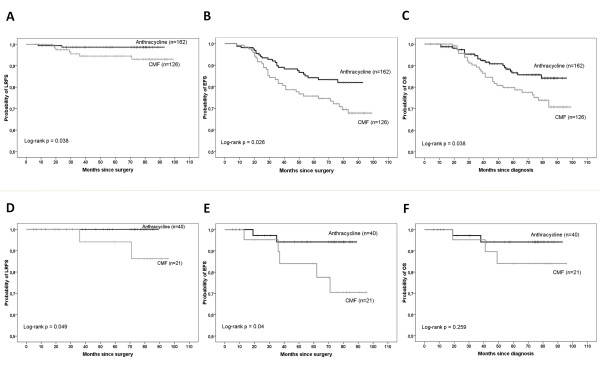
**Panels A, B, and C: Anthracycline (n = 162) compared to CMF (n = 126) in the subgroup of patients treated with ≥ 2 cycles of concurrent CT with RT: Kaplan-Meier estimates of LRFS (Panel A), EFS (Panel B), and OS (Panel C); Panels D, E, and F: Anthracycline (n = 40) compared to CMF (n = 21) in the subgroup of patients treated with BCT (n = 61): Kaplan-Meier estimates of LRFS (Panel D), EFS (Panel E), and OS (Panel F)**. LRFS = locoregional recurrence free survival; EFS = event free survival; OS = overall survival.

The median follow-up period was 73 months, 72 months, and 74 months, for LRFS, EFS, and OS respectively. Two patients developed loco-regional relapse anthracycline subgroup (n = 162) vs 7 patients in CMF subgroup (n = 126). The 5 years LRFS (type 1) rate was equal to 98.7% in anthracycline subgroup *vs *94.5% in CMF subgroup (log-rank *P = *.038) (Figure 3A). The 5 years EFS rate was 84.2% in anthracycline subgroup *vs *75.7% in CMF subgroup (log-rank *P *= .026) (Figure 3B). The 5 years OS rate was 86.6% in anthracycline subgroup vs 79.8% in CMF subgroup (log-rank *P *= .038) (Figure 3C).

#### 3.3 Outcomes in patients treated with BCT (n = 61)

The median follow-up period was 73 months, 73 months, and 75 months, for LRFS, EFS, and OS respectively. The 5 years LRFS rate (type 1) was equal to 100% in anthracycline subgroup *vs *94.1% in CMF subgroup (log-rank *P = *.049) (Figure 3D). The 5 years EFS rate was 94.2% in anthracycline subgroup *vs *84% in CMF subgroup (log-rank *P *= .04) (Figure 3E). The 5 years OS rate was 94.2% in anthracycline subgroup vs 84% in CMF subgroup (log-rank *P *= .259) (Figure 3F).

## Discussion

The main advantages of CCRT are: 1. delivering both treatments of CT and RT at same time; 2. biological synergy effect that can increase the efficacy of the treatment [34]. A CT based on liposomal doxorubicin, paclitaxel and vinorelbine, with concomitant RT in non operable and recurrent disease, was found to be of good efficacy and tolerability [34,35]. Reirradiation with concomitant CT was shown to have positive effect [34,36]. In adjuvant setting, CCRT has successfully been achieved by the concurrent use of CMF or taxanes with WBI [20-23].

Few prospective studies investigated CCRT using anthracycline regimen. The authors showed high rate of high grade skin toxicity, and more cardiac dysfunction, and concluded that this protocol cannot be used in practice [25-27]. However, in a recent phase I trial, the authors showed that PBI with concurrent dose dense doxorubicin and cyclophosphamide induced an acceptable hematologic and nonhematologic toxicity profile with likelihood of RD ≥ grade 2 is ≤ 11% [29]. These results were in favor of the use of CCRT based on anthracycline regimen and this is why we suggest the evaluation of concurrent versus sequential therapy in early stage breast cancer using anthracycline and modern radiation techniques. In our previous investigation we evaluated two concurrent protocols administered either with anthracycline regimen or with CMF in adjuvant setting and confirmed the beneficial effect of anthracycline based protocol in locoregional control with acceptable toxicity profile over the CMF [28].

After 74.5 months median follow-up, we found no statistical difference in the OS between the two therapeutic groups A and B, which can be explained by the fact that group A was characterized by poorer prognosis (more lymph node involvement). However, we showed a trend toward significance for EFS in favor of anthracycline (log-rank *P *= .057). In addition, when only the isolated locoregional events were considered, the LRFS at 5 years was significantly higher in group A than group B (98.7% vs 95.3%; log-rank *P *= .034). To confirm the beneficial effect of anthracycline we conducted subgroup analyses. The first subgroup concerned patients treated with ≥ 2 cycles of concurrent CT (n = 288 from A and B) and we showed that LRFS, EFS and OS were statistically higher in the anthracycline based treatment than in CMF. The second sub group concerned patients treated with BCT (n = 61 from A and B) and we showed that LRFS and EFS were statistically higher in anthracycline than in CMF. In addition, using the multivariate Cox proportional hazard regression models analysis, we confirmed that the anthracycline regimen favorably influenced the LRFS (type 1) (*P *= .062), the EFS (*P *= .007), and OS (*P *= .046) (table 2).

However, in adjuvant setting, it has been demonstrated that anthracycline and taxanes containing regimens decreased relapse and improved survival. Consequently, our results may be explained by the effect of anthracycline regimen and not by the use of CCRT [24,37].

Three recent randomized phase III trials were conducted to compare the sequential protocol to the concomitant protocol. In the first trial, 716 patients were treated by BCT and randomized into 2 groups (ARCOSEIN study) [38]. In the first group, the patients were treated by the FNC protocol (5-fluoro-uracil 500 mg/m2, mitoxantrone 12 mg/m^2 ^and cyclophosphamide 500 mg/m^2^) with concomitant RT. In the second group, the patients were treated by the FNC protocol followed by RT. Arcangely et al. [39], randomly assigned 206 patients (after quadrantectomy and axillary dissection) to concurrent or sequential treatments using CMF based CT. In the third trial, Rouessé et al. [40], randomly assigned 638 patients with prior breast surgery and positive axillary dissection (from which 416 were BCT) to receive CCRT (FNC protocol) or CT (fluorouracil, epirubicin, and cyclophosphamide protocol) followed by RT. No differences in 5-years LRFS, disease-free survival and OS were observed between the 2 treatment groups in the 3 trials. Nevertheless, in the ARCOSEIN study the authors identified a significant decrease in the risk of locoregional recurrence with CCRT for node-positive patients. Rouessé et al. [40], showed that concurrent treatment has a significantly better locoregional control in node-positive breast cancer after BCT.

In our study we found significantly higher locoregional control of the disease and a trend for a higher rate of 5 years EFS in anthracycline group vs CMF group. And we found a superior effect of concurrent use of anthracycline over CMF in terms of LRFS and EFS in the subgroup of patients treated with BCT. In addition, in the subgroup of patient treated with ≥ 2 cycles of concurrent CT, we confirmed the significant beneficial effect of the anthracycline in terms of LRFS, EFS and OS.

The main limitation of the 3 European trials was the use of CMF protocol and FNC protocol which have a safer toxicity profile when radiation treatment was used without the use of anthracycline and taxane regimens. CMF and FNC are older CT regimen used in the past; however more recently anthracyclines and taxanes containing regimens have become standard, and are demonstrated to have improved survival and local recurrence outcomes.

In ARCOSEIN study, moderate acute locoregional toxicities were found in the concomitant arm. Rouessé et al. [40] presented more frequent grade 2 skin toxicities in the concomitant arm, and more subclinical LVFE events at 1 year. In our study we showed more haematologic toxicity when the treatment was based on anthracycline with significantly more grade 3-4 neutropenia. Grade 2-3 anemia and grade ≥ 2 thrombopenia were more frequent in anthracycline group but the differences were not significant.

In our study, we found more respiratory symptoms and high grade (≥ grade 2) RD toxicity (4.8% vs 1.3%) in anthracycline group versus CMF group. We note however that dermatitis toxicity was not routinely evaluated like other adverse events as we would expect much higher dermatitis rates with concurrent taxane/radiation.

Our retrospective study showed different limitations, because it implicated potential bias in the choice of treatment. In addition to the lack of cardiac toxicity evaluation which constituted the major limitation (only 2% [n = 9] had assessment of cardiac function via LVEF measurement). However, 50% of the cases were left-sided breast cancers and would probability induce a high risk of cardiac toxicity.

## Conclusion

From the present 5 years nonrandomized investigation we concluded that the treatment based on anthracycline and concurrent RT reduced breast cancer relapse rate including locoregional relapses and significantly improved LRFS, EFS and OS in the patients receiving more than 1 cycle of concurrent CT. In multivariate analysis we confirmed that the anthracycline regimens had a positive effect on LRFS, EFS and OS. There were more hematologic and non hematologic toxicities in the anthracycline group. Anthracyclines and WBI cannot be administered concurrently before further investigations to determine efficacy and safety of anthracycline based treatment.

## Abbreviations

RT: radiotherapy; CT: chemotherapy; WBI; whole-breast-irradiation; CCRT: concurrent chemoradiotherapy; RD: radiation dermatitis; PBI: partial breast irradiation; CMF: cyclophosphamide 500 mg/m^2^, methotrexate 60 mg/m^2^, and 5-fluorouracil 500 mg/m^2^; EFS: event free survival; OS: overall survival; LRFS: locoregional recurrence free survival; AC60: doxorubicin 60 mg/m^2 ^and cyclophosphamide 600 mg/m^2^; FEC75: 5-fluorouracile 500 mg/m^2^, epirubicin 75 mg/m^2^, and cyclophosphamide 500 mg/m^2^; FAC50: 5-fluorouracile 500 mg/m^2^, doxorubicin 50 mg/m^2^, and cyclophosphamide 500 mg/m^2^; BCT: breast conservative therapy; LVFE: left ventricular fraction ejection; FNC: 5-fluoro-uracil 500 mg/m2, mitoxantrone 12 mg/m2 and cyclophosphamide 500 mg/m2.

## Competing interests

The authors declare that they have no competing interests.

## Authors' contributions

NI: conception and design, acquisition of data, analysis and interpretation of data, statistical analysis, literature review, drafting the manuscript and revising it critically for important intellectual content; SE: acquisition and analysis of data; IL: acquisition of data; LB: acquisition of data; RB: review of finale manuscript; HA: help in statistical analysis, RA: acquisition of data; NB: review of finale manuscript; BEKEG: review of finale manuscript; OE: conception and design; YS: review of finale manuscript; MI: involved in drafting the manuscript; HE: conception, design and review of final manuscript.

All authors read and approved the final manuscript.

## References

[B1] Pierce LJ Lichter AS Defining the role of post-mastectomy radiotherapy: The new evidence Oncology (Williston Park) 1996 10 991 1002 8837118

[B2] Fowble B Postmastectomy radiotherapy: Then and now Oncology (Williston Park) 1997 11 213 239 9057176

[B3] Overgaard M Hansen PS Overgaard J Rose C Andersson M Bach F Kjaer M Gadeberg CC Mouridsen HT Jensen MB Zedeler K Postoperative radiotherapy in high-risk premenopausal women with breast cancer who receive adjuvant chemotherapy N Engl J Med 1997 337 949 955 10.1056/NEJM199710023371401 9395428

[B4] Overgaard M Jensen MB Overgaard J Hansen PS Rose C Andersson M Kamby C Kjaer M Gadeberg CC Rasmussen BB Blichert-Toft M Mouridsen HT Postoperative radiotherapy in high-risk postmenopausal breast-cancer patients given adjuvant tamoxifen: Danish Breast Cancer Cooperative Group DBCG 82c randomized trial Lancet 1999 353 1641 1648 10.1016/S0140-6736(98)09201-0 10335782

[B5] Ragaz J Olivotto I Spinelli JJ Phillips N Jackson SM Wilson KS Knowling MA Coppin CM Weir L Gelmon K Le N Durand R Coldman AJ Manji M Locoregional radiation therapy in patients with high-risk breast cancer receiving adjuvant chemotherapy: 20-year results of the British Columbia randomized trial J Natl Cancer Inst 2005 97 116 126 10.1093/jnci/djh297 15657341

[B6] Early Breast Cancer Trialists' Collaborative Group Effects of radiotherapy and surgery in early breast cancer: An overview of the randomized trials N Engl J Med 1995 333 1444 1455 10.1056/NEJM199511303332202 7477144

[B7] Early Breast Cancer Trialists' Collaborative Group Favourable and unfavourable effects on long-term survival of radiotherapy for early breast cancer: An overview of the randomized trials Lancet 2000 355 1757 1770 10.1016/S0140-6736(00)02263-7 10832826

[B8] Veronesi U Marubini E Mariani L Galimberti V Luini A Veronesi P Salvadori B Zucali R Radiotherapy after breast-conserving surgery in small breast carcinoma: Longterm results of a randomized trial Ann Oncol 2001 12 997 1003 10.1023/A:1011136326943 11521809

[B9] Fisher B Anderson S Bryant J Margolese RG Deutsch M Fisher ER Jeong JH Wolmark N Twenty-years follow-up of a randomized trial comparing total mastectomy, lumpectomy, and lumpectomy plus irradiation for the treatment of invasive breast cancer N Engl J Med 2002 347 1233 1241 10.1056/NEJMoa022152 12393820

[B10] Fisher B Carbone P Economou SG Frelick R Glass A Lerner H Redmond C Zelen M Band P Katrych DL Wolmark N Fisher ER Phenylalanine mustard (L-PAM) in the management of primary breast cancer: a report of early findings N Engl J Med 1975 292 117 122 10.1056/NEJM197501162920301 1105174

[B11] Bonadonna G Brusamolino E Valagussa P Rossi A Brugnatelli L Brambilla C De Lena M Tancini G Bajetta E Musumeci R Veronesi U Combination chemotherapy as an adjuvant treatment in operable breast cancer N Engl J Med 1976 294 405 410 10.1056/NEJM197602192940801 1246307

[B12] Early Breast Cancer Trialists' Collaborative Group Systemic treatment of early breast cancer by hormonal cytotoxic, or immune therapy: 133 randomized trials involving 31,000 recurrences and 24,000 deaths along 75,000 women Lancet 1992 339 1 15 1345950

[B13] Recht A Come SE Henderson IC Gelman RS Silver B Hayes DF Shulman LN Harris JR The sequencing of chemotherapy and radiation therapy after conservative surgery for early-stage breast cancer N Engl J Med 1996 334 1356 1361 10.1056/NEJM199605233342102 8614420

[B14] Vujovic O Perera F Dar AR Stitt L Yu E Voruganti SM Truong PT Does delay in breast irradiation following conservative breast surgery in nodenegative cancer patients have an impact on risk of recurrence? Int J Radiat Oncol Biol Phys 1998 40 869 874 9531372 10.1016/s0360-3016(97)00922-x

[B15] Buchholz TA Hunt KK Amosson CM Tucker SL Strom EA McNeese MD Buzdar AU Singletary SE Hortobagyi GN Sequencing of chemotherapy and radiation in lymph node-negative breast cancer Cancer J Sci Am 1999 5 159 164 10367172

[B16] Leonard CE Wood ME Zhen B Rankin J Waitz DA Norton L Howell K Sedlacek S Does administration of chemotherapy before radiotherapy in breast cancer patients treated with conservative surgery negatively impact local control? J Clin Oncol 1995 13 2906 2915 8523054 10.1200/JCO.1995.13.12.2906

[B17] Hartsell WF Recine DC Griem KL Murthy AK Delaying the initiation of intact breast irradiation for patients with lymph node positive breast cancer increases the risk of local recurrence Cancer 1995 76 2497 2503 10.1002/1097-0142(19951215)76:12<2497::AID-CNCR2820761214>3.0.CO;2-6 8625076

[B18] Buchholz TA Austin-Seymour MM Moe RE Ellis GK Livingston RB Pelton JG Griffin TW Effect of delay in radiation in the combined modality treatment of breast cancer Int J Radiat Oncol Biol Phys 1993 26 23 35 8482628 10.1016/0360-3016(93)90169-v

[B19] Recht A Come SE Gelman RS Goldstein M Tishler S Gore SM Abner AL Vicini FA Silver B Connolly JL Integration of conservative surgery, radiotherapy and chemotherapy for the treatment of early-stage, node-positive breast cancer: Sequencing, timing, and outcome J Clin Oncol 1991 9 1662 1667 1875223 10.1200/JCO.1991.9.9.1662

[B20] Dubey AK Recht A Come S Shulman L Harris J Why and how to combine chemotherapy and radiation therapy in breast cancer patients Recent Results Cancer Res 1998 152 247 254 9928562 10.1007/978-3-642-45769-2_23

[B21] Dubey A Recht A Come SE Gelman RS Silver B Harris JR Shulman LN Concurrent CMF and radiation therapy for early stage breast cancer: Results of a pilot study Int J Radiat Oncol Biol Phys 1999 45 877 884 10571193 10.1016/s0360-3016(99)00295-3

[B22] Faul C Brufsky A Gerszten K Flickinger J Kunschner A Jacob H Vogel V Concurrent sequencing of full-dose CMF chemotherapy and radiation therapy in early breast cancer has no effect on treatment delivery Eur J Cancer 2003 39 763 768 10.1016/S0959-8049(02)00834-1 12651201

[B23] Formenti SC Volm M Skinner KA Spicer D Cohen D Perez E Bettini AC Groshen S Gee C Florentine B Press M Danenberg P Muggia F Preoperative twice-weekly paclitaxel with concurrent radiation therapy followed by surgery and postoperative doxorubicin-based chemotherapy in locally advanced breast cancer: A phase I/II trial J Clin Oncol 2003 21 864 870 10.1200/JCO.2003.06.132 12610186

[B24] Effects of chemotherapy and hormonal therapy for early breast cancer on recurrence and 15-year survival: an overview of the randomised trials. Early Breast Cancer Trialists' Collaborative Group (EBCTCG) Lancet 2005 20;365 9472 1687 717 10.1016/S0140-6736(05)66544-0 15894097

[B25] Hoogenraad W Franssen J van Turnhout J Enhanced toxicity of radiotherapy due to epirubicin containing adjuvant chemotherapy in breast carcinoma patients Radiother Oncol 1992 24 S42 (abstr 158)

[B26] Fiets WE van Helvoirt RP Nortier JW van der Tweel I Struikmans H Acute toxicity of concurrent adjuvant radiotherapy and chemotherapy (CMF or AC) in breast cancer patients: A prospective, comparative, non-randomised study Eur J Cancer 2003 39 1081 1088 10.1016/S0959-8049(03)00178-3 12736107

[B27] Clements IP Davis BJ Wiseman GA Systolic and diastolic cardiac dysfunction early after the initiation of doxorubicin therapy: Significance of gender and concurrent mediastinal radiation Nucl Med Commun 2002 23 521 527 10.1097/00006231-200206000-00003 12029206

[B28] Ismaili N Mellas N Masbah O Elmajjaoui S Arifi S Bekkouch I Ahid S Bazid Z Tazi MA Erraki A El Mesbahi O Benjaafar N El Gueddari Bel K Ismaili M Afqir S Errihani H Concurrent chemoradiotherapy in adjuvant treatment of breast cancer Radiat Oncol 2009 4 12 10.1186/1748-717X-4-12 19351405 PMC2679760

[B29] Zellars RC Stearns V Frassica D Asrari F Tsangaris T Myers L DiPasquale S Lange JR Jacobs LK Emens LA Armstrong DK Fetting JH Garrett-Mayer E Davidson NE Wolff AC Feasibility trial of partial breast irradiation with concurrent dose-dense doxorubicin and cyclophosphamide in early-stage breast cancer J Clin Oncol 2009 27 2816 22 10.1200/JCO.2008.20.0139 19332718 PMC4859211

[B30] Ismaili N Errihani H Anthracycline and concurrent radiotherapy significantly reduced loco-regional breast cancer relapse rate [abstract] EJC supplements 2010 8 72

[B31] Greene FL Page DL Fleming ID AJCC cancer staging manual 2002 6 New York: Springer-Verglag

[B32] Kaplan EL Meier P Nonparametric estimation from incomplete observations J Am Stat Assoc 1958 53 457 481 10.2307/2281868

[B33] Cox DR Regression models and life-tables J R Stat Soc B 1972 34 187 202

[B34] Kouloulias VE Dardoufas CE Kouvaris JR Gennatas CS Polyzos AK Gogas HJ Sandilos PH Uzunoglu NK Malas EG Vlahos LJ Liposomal doxorubicin in conjunction with reirradiation and local hyperthermia treatment in recurrent breast cancer: a phase I/II trial Clin Cancer Res 2002 8 374 82 11839652

[B35] Kao J Conzen SD Jaskowiak NT Song DH Recant W Singh R Masters GA Fleming GF Heimann R Concomitant radiation therapy and paclitaxel for unresectable locally advanced breast cancer: results from two consecutive phase I/II trials Int J Radiat Oncol Biol Phys 2005 61 4 1045 53 15752883 10.1016/j.ijrobp.2004.07.714

[B36] Würschmidt F Dahle J Petersen C Wenzel C Kretschmer M Bastian C Reirradiation of recurrent breast cancer with and without concurrent chemotherapy Radiat Oncol 2008 3 28 10.1186/1748-717X-3-28 18801165 PMC2556652

[B37] Bedard PL Di Leo A Piccart-Gebhart MJ Taxanes: optimizing adjuvant chemotherapy for early-stage breast cancer Nat Rev Clin Oncol 2010 7 1 22 36 10.1038/nrclinonc.2009.186 19997076

[B38] Toledano A Azria D Garaud P Fourquet A Serin D Bosset JF Miny-Buffet J Favre A Le Floch O Calais G Phase III trial of concurrent or sequential adjuvant chemoradiotherapy after conservative surgery for early-stage breast cancer: Final results of the ARCOSEIN trial J Clin Oncol 2007 25 405 10 10.1200/JCO.2006.07.8576 17264336

[B39] Arcangeli G Pinnaro P Rambone R Giannarelli D Benassi M A phase III randomized study on the sequence of radiotherapy and chemotherapy in the conservative management of early-stage breast cancer Int J Radiat Oncol Biol Phys 2006 64 161 167 16226397 10.1016/j.ijrobp.2005.06.040

[B40] Rouessé J De la Lande B Bertheault-Cvitkovic F Serin D Graïc Y Combe M Leduc B Lucas V Demange L Nguyen TD Castèra D Krzisch C Villet R Mouret-Fourme E Garbay JR Noguès C Centre René Huguenin Breast Cancer Group A phase III randomized trial comparing adjuvant concomitant chemoradiotherapy versus standard adjuvant chemotherapy followed by radiotherapy in operable node-positive breast cancer: Final results Int J Radiat Oncol Biol Phys 2006 64 1072 1080 16504757 10.1016/j.ijrobp.2005.10.011

